# 4-(4-Bromo­phen­yl)-7,7-dimethyl-2-methyl­amino-3-nitro-7,8-di­hydro-4*H*-chromen-5(6*H*)-one including an unknown solvate

**DOI:** 10.1107/S1600536814007983

**Published:** 2014-04-18

**Authors:** S. Antony Inglebert, Jayabal Kamalraja, K. Sethusankar, Gnanasambandam Vasuki

**Affiliations:** a Sri Ram Engineering College, Chennai 602 024, India; b Department of Chemistry, Pondichery University, Pondichery 605 014, India; c Department of Physics, RKM Vivekananda College (Autonomous), Chennai 600 004, India

## Abstract

In the title compound, C_18_H_19_BrN_2_O_4_, the chromene unit is not quite planar (r.m.s. deviation = 0.199 Å), with the methyl C atoms lying 0.027 (4) and 1.929 (4) Å from the mean plane of the chromene unit. The six-membered carbocyclic ring of the chromene moiety adopts an envelope conformation, with the dimethyl-substituted C atom as the flap. The methyl­amine and nitro groups are slightly twisted from the chromene moiety, with C—N—C—O and O—N—C—C torsion angles of 2.7 (4) and −0.4 (4)°, respectively. The dihedral angle between the mean plane of the chromene unit and the benzene ring is 85.61 (13)°. An intra­molecular N—H⋯O hydrogen bond generates an *S*(6) ring motif, which stabilizes the mol­ecular conformation. In the crystal, mol­ecules are linked *via* N—H⋯O hydrogen bonds, forming hexa­gonal rings lying parallel to the *ab* plane. A region of disordered electron density, most probably disordered ethanol solvent mol­ecules, occupying voids of *ca* 432 Å^3^ for an electron count of 158, was treated using the SQUEEZE routine in *PLATON* [Spek (2009 ▶). *Acta Cryst*. D**65**, 148–155]. Their formula mass and unit-cell characteristics were not taken into account during refinement.

## Related literature   

For the biological and pharmacological properties of chromene and chromene derivatives, see: Thomas & Zachariah (2013 ▶). For graph-set notation, see: Bernstein *et al.* (1995 ▶). For ring puckering parameters, see: Cremer & Pople (1975 ▶). For a related structure, see: Narayanan *et al.* (2013 ▶).
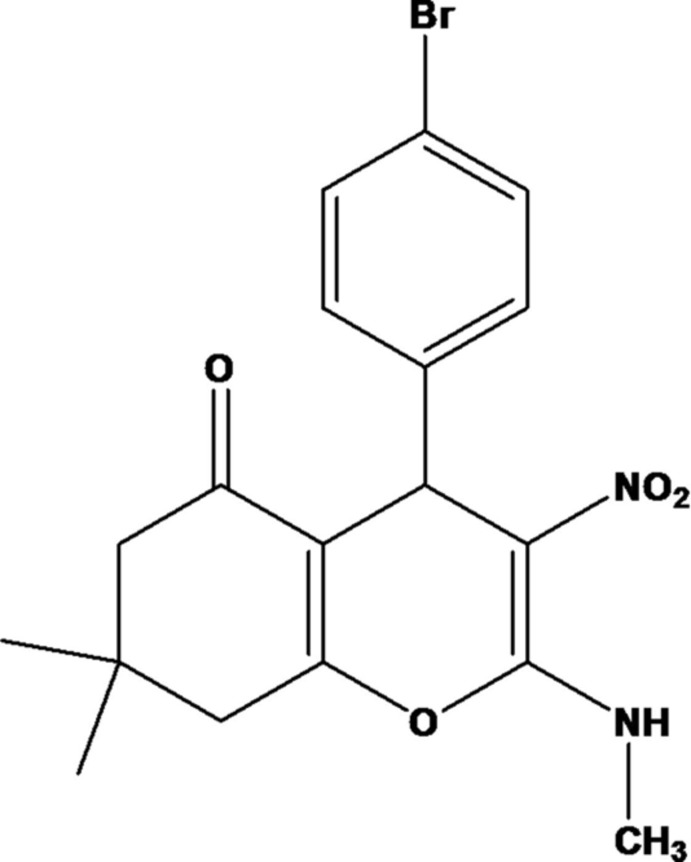


## Experimental   

### 

#### Crystal data  

C_18_H_19_BrN_2_O_4_
*M* *_r_* = 407.26
Trigonal, 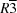

*a* = 24.2105 (13) Å
*c* = 15.7745 (9) Å
*V* = 8007.4 (8) Å^3^
*Z* = 18
Mo *K*α radiation
μ = 2.34 mm^−1^
*T* = 293 K
0.35 × 0.30 × 0.30 mm

#### Data collection  

Bruker Kappa APEXII CCD diffractometer
Absorption correction: multi-scan (*SADABS*; Bruker, 2008 ▶) *T*_min_ = 0.446, *T*_max_ = 0.496
25281 measured reflections
3206 independent reflections
2565 reflections with *I* > 2σ(*I*)
*R*_int_ = 0.035

#### Refinement  

*R*[*F* ^2^ > 2σ(*F* ^2^)] = 0.038
*wR*(*F* ^2^) = 0.105
*S* = 1.09
3206 reflections
233 parameters
H atoms treated by a mixture of independent and constrained refinement
Δρ_max_ = 0.68 e Å^−3^
Δρ_min_ = −0.61 e Å^−3^

### 

Data collection: *APEX2* (Bruker, 2008 ▶); cell refinement: *SAINT* (Bruker, 2008 ▶); data reduction: *SAINT*; program(s) used to solve structure: *SHELXS97* (Sheldrick, 2008 ▶); program(s) used to refine structure: *SHELXL97* (Sheldrick, 2008 ▶); molecular graphics: *PLATON* (Spek, 2009 ▶) and *Mercury* (Macrae *et al.*, 2008 ▶); software used to prepare material for publication: *SHELXL97* and *PLATON* (Spek, 2009 ▶).

## Supplementary Material

Crystal structure: contains datablock(s) global, I. DOI: 10.1107/S1600536814007983/su2714sup1.cif

Structure factors: contains datablock(s) I. DOI: 10.1107/S1600536814007983/su2714Isup2.hkl

Supporting information file. DOI: 10.1107/S1600536814007983/su2714Isup3.cml

CCDC reference: 996468

Additional supporting information:  crystallographic information; 3D view; checkCIF report

## Figures and Tables

**Table 1:** Hydrogen-bond geometry (Å, °)

*D*—H⋯*A*	*D*—H	H⋯*A*	*D*⋯*A*	*D*—H⋯*A*
N2—H2*N*⋯O3	0.83 (3)	2.00 (3)	2.618 (3)	130 (3)
N2—H2*N*⋯O4^i^	0.83 (3)	2.38 (3)	2.969 (3)	129 (3)

Symmetry code: (i) 
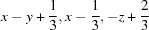
.

**Fig. 1.**
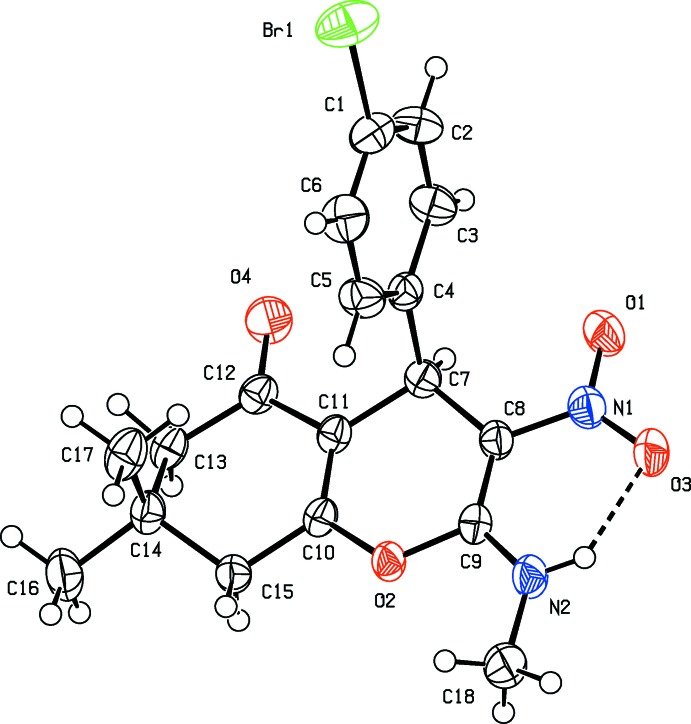
A view of the molecular structure of the title molecule, with atom labelling. Displacement ellipsoids drawn at the 30% probability level. The intramolecular N—H···O hydrogen bond is shown as a dashed line (see Table 1 for details).

**Fig. 2.**
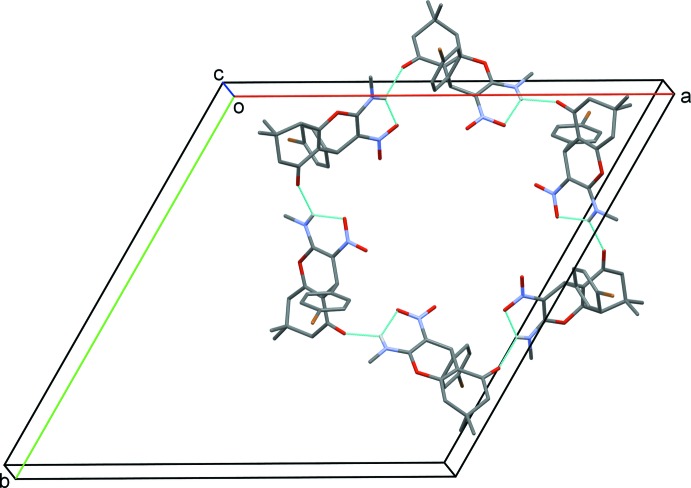
A partial view along the c axis of the crystal packing of the title compound. Hydrogen bonds are shown as dashed lines (see Table 1 for details; H atoms not involved in hydrogen bonding have been omitted for clarity).

## supplementary crystallographic information

### 1. Comment

Chromene constitutes the basic backbone of various types of polyphenols and is widely found in natural alkaloids, tocopherols, flavonoids and anthocyanins. Natural and synthetic chromene derivatives possess important biological activities such as antitumor, antispasmolytic, antivascular, anticancer, anti-HIV, estrogenic and herbicidal activity. They also plays an important role in the production of highly effective fluorescent dyes for synthetic fibers, daylight fluorescent pigments and electrophotographic and electroluminescent devices (Thomas *et al.*, 2013).

The title compound, Fig. 1, consists of a chromene unit connected to a bromophenyl ring at C7, a nitro group at C8, a methyl amine group at C9, an oxygen atom at C12 and a dimethyl group at C14. The mean plane of the chromene unit (O2/C7–C15) is almost normal to the benzene ring (C1–C6), with a dihedral angle of 85.61 (13)°. The mean plane of the chromene unit makes dihedral angles of 7.25 (21) and 2.89 (21)° with the nitro and methylamine groups, respectively.

The six membered carbocyclic ring (C10–C15) of the chromene moiety has an envelope conformation with puckering parameters (Cremer & Pople, 1975), of Puckering Amplitude (Q) = 0.459 (3) Å, θ = 124.1 (4) °, φ = 57.4 (5) °. Atom C14 deviates by -0.324 (3) Å from the mean plane passing through the other five C ring atoms. The sum of the angles around atom N1 (359.9 °) is in accordance with *sp^2^* hybridization. The amine group nitrogen atoms, N1 and N2, deviate by -0.156 (2) and -0.0153 (3) Å from the mean plane of the chromene unit. The bromine atom, Br1, deviates from the benzene ring (C1–C6) by 0.0526 (5) Å. The methyl amine group attached to C9 is coplanar with the chromene unit as indicated by the torsion angle C18-N2-C9-O2 = 2.7 (4)°. The nitro group is also coplanar to the chromene unit, as indicated by the torsion angles O1-N1-C8-C7 = -5.5 (3)° and O3-N1-C8-C9 = -0.4 (4)°, respectively. The molecular structure is characterized by an intramolecular N—H···O hydrogen bond, which generates an *S(6)* ring motif (Bernstein *et al.*, 1995). The title compound exhibits structural similarities with the related structure, 4-(4-Bromophenyl)-2-methylamino-3-nitro-5,6,7,8-tetrahydro-4*H*-chromen-5-one (Narayanan *et al.*, 2013).

The crystal packing is stabilized by intermolecular N—H···O hydrogen bonds forming hexagonal rings centered about a threefold rotation axis and lying parallel to the *ab* plane (Fig. 2 and Table 1). The amide N1 atom is involved in both intra and intermolecular hydrogen bonding, having a bifurcated character (Table 1).

### 2. Experimental

A solution of the 4-bromobenzaldehyde (1.0 mmol), 5,5- dimethylcyclohexane-1,3-dione (1.0 mmol), NMSM (1.0 mmol), and *L*-proline (0.2 equiv) in EtOH (2 ml) was stirred for the 2.3 h until the reaction was complete as indicated by TLC. The product obtained was filtered and washed with EtOH (2 ml) to remove the excess base and other impurities. Finally, the products were recrystallized from EtOH yielding block-like colourless crystals.

### 3. Refinement

The NH H atom was located in a difference Fourier map and freely refined. The C-bound H atoms were placed in idealized positions and allowed to ride on the parent atoms: C—H = 0.93 - 0.97 Å with *U*_iso_(H)= 1.5 *U*_eq_(*C*-methyl) and = 1.2*U*_eq_(C) for other H atoms. A region of disordered electron density, most probably disordered ethanol solvent molecules, occupying voids of *ca* 432 Å^3^ for an electron count of 158, was treated using the SQUEEZE routine in *PLATON* [Spek (2009). Acta Cryst. D65, 148–155]. The formula mass and unit-cell characteristics were not taken into account during refinement.

### Crystal data

**Table:**

C_18_H_19_BrN_2_O_4_	*D*_x_ = 1.520 Mg m^−^^3^
*M**_r_* = 407.26	Mo *K*α radiation, λ = 0.71073 Å
Trigonal, *R*3	Cell parameters from 3206 reflections
Hall symbol: -R 3	θ = 2.3–25.2°
*a* = 24.2105 (13) Å	µ = 2.34 mm^−^^1^
*c* = 15.7745 (9) Å	*T* = 293 K
*V* = 8007.4 (8) Å^3^	Block, colourless
*Z* = 18	0.35 × 0.30 × 0.30 mm
*F*(000) = 3744	

### Data collection

**Table:**

Bruker Kappa APEXII CCD diffractometer	3206 independent reflections
Radiation source: fine-focus sealed tube	2565 reflections with *I* > 2σ(*I*)
Graphite monochromator	*R*_int_ = 0.035
ω and φ scan	θ_max_ = 25.2°, θ_min_ = 2.3°
Absorption correction: multi-scan (*SADABS*; Bruker, 2008)	*h* = −28→29
*T*_min_ = 0.446, *T*_max_ = 0.496	*k* = −29→28
25281 measured reflections	*l* = −12→18

### Refinement

**Table:**

Refinement on *F*^2^	Primary atom site location: structure-invariant direct methods
Least-squares matrix: full	Secondary atom site location: difference Fourier map
*R*[*F*^2^ > 2σ(*F*^2^)] = 0.038	Hydrogen site location: inferred from neighbouring sites
*wR*(*F*^2^) = 0.105	H atoms treated by a mixture of independent and constrained refinement
*S* = 1.09	*w* = 1/[σ^2^(*F*_o_^2^) + (0.0569*P*)^2^ + 9.8133*P*] where *P* = (*F*_o_^2^ + 2*F*_c_^2^)/3
3206 reflections	(Δ/σ)_max_ < 0.001
233 parameters	Δρ_max_ = 0.68 e Å^−^^3^
0 restraints	Δρ_min_ = −0.61 e Å^−^^3^

### Special details

**Table:**

Geometry. All e.s.d.'s (except the e.s.d. in the dihedral angle between two l.s. planes) are estimated using the full covariance matrix. The cell e.s.d.'s are taken into account individually in the estimation of e.s.d.'s in distances, angles and torsion angles; correlations between e.s.d.'s in cell parameters are only used when they are defined by crystal symmetry. An approximate (isotropic) treatment of cell e.s.d.'s is used for estimating e.s.d.'s involving l.s. planes.
Refinement. Refinement of *F*^2^ against ALL reflections. The weighted *R*-factor *wR* and goodness of fit *S* are based on *F*^2^, conventional *R*-factors *R* are based on *F*, with *F* set to zero for negative *F*^2^. The threshold expression of *F*^2^ > σ(*F*^2^) is used only for calculating *R*-factors(gt) *etc*. and is not relevant to the choice of reflections for refinement. *R*-factors based on *F*^2^ are statistically about twice as large as those based on *F*, and *R*- factors based on ALL data will be even larger.

### Fractional atomic coordinates and isotropic or equivalent isotropic displacement parameters (Å^2^)

**Table:**

	*x*	*y*	*z*	*U*_iso_*/*U*_eq_	
Br1	0.212132 (19)	0.139268 (18)	0.68871 (2)	0.06222 (17)	
O1	0.43022 (10)	0.17889 (10)	0.40027 (13)	0.0472 (5)	
O2	0.26619 (8)	0.05160 (8)	0.21543 (11)	0.0321 (4)	
O3	0.42426 (10)	0.09139 (10)	0.35374 (13)	0.0447 (5)	
O4	0.27248 (10)	0.24234 (10)	0.29435 (14)	0.0504 (5)	
N1	0.40219 (10)	0.12873 (11)	0.35810 (13)	0.0354 (5)	
N2	0.32782 (12)	0.01391 (11)	0.25833 (16)	0.0374 (6)	
H2N	0.3582 (17)	0.0173 (16)	0.287 (2)	0.056 (10)*	
C1	0.24640 (15)	0.14478 (14)	0.57850 (17)	0.0399 (7)	
C2	0.30235 (15)	0.19826 (14)	0.55683 (18)	0.0439 (7)	
H2	0.3238	0.2310	0.5958	0.053*	
C3	0.32632 (14)	0.20269 (13)	0.47641 (17)	0.0390 (7)	
H3	0.3643	0.2389	0.4613	0.047*	
C4	0.29519 (12)	0.15445 (12)	0.41766 (16)	0.0301 (6)	
C5	0.23957 (13)	0.10049 (13)	0.44189 (18)	0.0397 (7)	
H5	0.2186	0.0672	0.4035	0.048*	
C6	0.21442 (15)	0.09504 (15)	0.52256 (19)	0.0455 (7)	
H6	0.1769	0.0587	0.5384	0.055*	
C7	0.32208 (12)	0.16060 (12)	0.32833 (15)	0.0293 (6)	
H7A	0.3573	0.2044	0.3209	0.035*	
C8	0.34739 (12)	0.11581 (12)	0.31480 (15)	0.0297 (6)	
C9	0.31590 (12)	0.06113 (12)	0.26513 (16)	0.0297 (6)	
C10	0.24637 (12)	0.09617 (12)	0.21277 (15)	0.0278 (6)	
C11	0.27201 (11)	0.14784 (12)	0.26209 (15)	0.0281 (5)	
C12	0.24891 (12)	0.19332 (12)	0.25304 (16)	0.0313 (6)	
C13	0.19699 (12)	0.17837 (12)	0.18972 (17)	0.0336 (6)	
H13A	0.2165	0.1994	0.1367	0.040*	
H13B	0.1714	0.1963	0.2099	0.040*	
C14	0.15270 (12)	0.10710 (13)	0.17229 (17)	0.0335 (6)	
C15	0.19391 (12)	0.07738 (13)	0.15019 (17)	0.0321 (6)	
H15A	0.1674	0.0313	0.1494	0.039*	
H15B	0.2119	0.0912	0.0940	0.039*	
C16	0.10934 (15)	0.09891 (16)	0.0969 (2)	0.0502 (8)	
H16A	0.0811	0.0543	0.0866	0.075*	
H16B	0.1350	0.1182	0.0475	0.075*	
H16C	0.0848	0.1191	0.1095	0.075*	
C17	0.11220 (14)	0.07477 (14)	0.2504 (2)	0.0458 (7)	
H17A	0.0850	0.0301	0.2393	0.069*	
H17B	0.0866	0.0937	0.2636	0.069*	
H17C	0.1395	0.0802	0.2976	0.069*	
C18	0.29496 (15)	−0.04065 (14)	0.2025 (2)	0.0477 (8)	
H18A	0.3047	−0.0730	0.2189	0.072*	
H18B	0.3086	−0.0278	0.1452	0.072*	
H18C	0.2498	−0.0573	0.2066	0.072*	

### Atomic displacement parameters (Å^2^)

**Table:**

	*U*^11^	*U*^22^	*U*^33^	*U*^12^	*U*^13^	*U*^23^
Br1	0.0846 (3)	0.0630 (3)	0.0458 (2)	0.0419 (2)	0.02115 (17)	0.00610 (15)
O1	0.0358 (11)	0.0452 (13)	0.0568 (13)	0.0174 (10)	−0.0149 (10)	−0.0151 (10)
O2	0.0316 (10)	0.0282 (10)	0.0418 (10)	0.0190 (8)	−0.0093 (8)	−0.0064 (8)
O3	0.0418 (12)	0.0528 (13)	0.0520 (12)	0.0330 (11)	−0.0116 (9)	−0.0050 (10)
O4	0.0536 (13)	0.0335 (12)	0.0689 (14)	0.0254 (10)	−0.0152 (11)	−0.0155 (10)
N1	0.0303 (12)	0.0409 (14)	0.0338 (12)	0.0169 (12)	−0.0007 (10)	0.0019 (10)
N2	0.0382 (14)	0.0366 (14)	0.0460 (13)	0.0254 (12)	−0.0108 (11)	−0.0049 (11)
C1	0.0492 (18)	0.0427 (17)	0.0363 (14)	0.0294 (15)	0.0069 (13)	0.0028 (12)
C2	0.0535 (19)	0.0352 (16)	0.0377 (15)	0.0183 (15)	−0.0045 (14)	−0.0096 (12)
C3	0.0381 (16)	0.0269 (15)	0.0414 (15)	0.0082 (13)	−0.0028 (12)	−0.0038 (11)
C4	0.0296 (14)	0.0278 (14)	0.0359 (13)	0.0168 (12)	−0.0028 (11)	−0.0024 (11)
C5	0.0357 (16)	0.0311 (15)	0.0422 (15)	0.0091 (13)	−0.0017 (12)	−0.0066 (12)
C6	0.0372 (17)	0.0399 (17)	0.0494 (17)	0.0119 (14)	0.0076 (13)	0.0037 (14)
C7	0.0261 (13)	0.0250 (13)	0.0344 (13)	0.0109 (11)	−0.0002 (10)	−0.0011 (11)
C8	0.0241 (13)	0.0335 (14)	0.0315 (13)	0.0143 (12)	−0.0012 (10)	0.0003 (11)
C9	0.0244 (13)	0.0325 (14)	0.0337 (13)	0.0153 (12)	0.0009 (10)	0.0022 (11)
C10	0.0259 (13)	0.0253 (13)	0.0345 (13)	0.0145 (11)	0.0022 (10)	0.0019 (10)
C11	0.0238 (13)	0.0261 (14)	0.0343 (13)	0.0125 (11)	0.0019 (10)	0.0011 (11)
C12	0.0302 (14)	0.0252 (14)	0.0386 (14)	0.0140 (12)	0.0026 (11)	−0.0001 (11)
C13	0.0338 (15)	0.0304 (14)	0.0423 (15)	0.0203 (13)	0.0041 (12)	0.0037 (12)
C14	0.0286 (14)	0.0297 (14)	0.0445 (15)	0.0164 (12)	−0.0025 (12)	0.0002 (11)
C15	0.0308 (14)	0.0314 (14)	0.0371 (14)	0.0177 (12)	−0.0062 (11)	−0.0057 (11)
C16	0.0410 (17)	0.0495 (19)	0.067 (2)	0.0275 (16)	−0.0180 (15)	−0.0085 (16)
C17	0.0321 (16)	0.0385 (17)	0.0652 (19)	0.0164 (14)	0.0095 (14)	0.0054 (14)
C18	0.0516 (19)	0.0396 (17)	0.0617 (19)	0.0302 (16)	−0.0143 (15)	−0.0141 (15)

### Geometric parameters (Å, º)

**Table:**

Br1—C1	1.902 (3)	C7—H7A	0.9800
O1—N1	1.247 (3)	C8—C9	1.392 (4)
O2—C9	1.356 (3)	C10—C11	1.334 (3)
O2—C10	1.384 (3)	C10—C15	1.489 (3)
O3—N1	1.261 (3)	C11—C12	1.470 (4)
O4—C12	1.217 (3)	C12—C13	1.501 (4)
N1—C8	1.382 (3)	C13—C14	1.534 (4)
N2—C9	1.316 (3)	C13—H13A	0.9700
N2—C18	1.450 (4)	C13—H13B	0.9700
N2—H2N	0.83 (3)	C14—C17	1.525 (4)
C1—C2	1.369 (4)	C14—C16	1.532 (4)
C1—C6	1.377 (4)	C14—C15	1.534 (4)
C2—C3	1.377 (4)	C15—H15A	0.9700
C2—H2	0.9300	C15—H15B	0.9700
C3—C4	1.382 (4)	C16—H16A	0.9600
C3—H3	0.9300	C16—H16B	0.9600
C4—C5	1.381 (4)	C16—H16C	0.9600
C4—C7	1.528 (3)	C17—H17A	0.9600
C5—C6	1.388 (4)	C17—H17B	0.9600
C5—H5	0.9300	C17—H17C	0.9600
C6—H6	0.9300	C18—H18A	0.9600
C7—C8	1.504 (4)	C18—H18B	0.9600
C7—C11	1.511 (3)	C18—H18C	0.9600
			
C9—O2—C10	120.62 (19)	C10—C11—C7	123.0 (2)
O1—N1—O3	120.5 (2)	C12—C11—C7	118.7 (2)
O1—N1—C8	118.5 (2)	O4—C12—C11	120.7 (2)
O3—N1—C8	120.9 (2)	O4—C12—C13	121.3 (2)
C9—N2—C18	125.6 (2)	C11—C12—C13	118.1 (2)
C9—N2—H2N	116 (2)	C12—C13—C14	114.9 (2)
C18—N2—H2N	119 (2)	C12—C13—H13A	108.6
C2—C1—C6	121.7 (3)	C14—C13—H13A	108.6
C2—C1—Br1	119.0 (2)	C12—C13—H13B	108.6
C6—C1—Br1	119.3 (2)	C14—C13—H13B	108.6
C1—C2—C3	118.9 (3)	H13A—C13—H13B	107.5
C1—C2—H2	120.6	C17—C14—C16	109.7 (2)
C3—C2—H2	120.6	C17—C14—C15	110.2 (2)
C2—C3—C4	121.4 (3)	C16—C14—C15	109.1 (2)
C2—C3—H3	119.3	C17—C14—C13	109.9 (2)
C4—C3—H3	119.3	C16—C14—C13	109.5 (2)
C5—C4—C3	118.4 (2)	C15—C14—C13	108.4 (2)
C5—C4—C7	120.9 (2)	C10—C15—C14	111.2 (2)
C3—C4—C7	120.7 (2)	C10—C15—H15A	109.4
C4—C5—C6	121.2 (3)	C14—C15—H15A	109.4
C4—C5—H5	119.4	C10—C15—H15B	109.4
C6—C5—H5	119.4	C14—C15—H15B	109.4
C1—C6—C5	118.4 (3)	H15A—C15—H15B	108.0
C1—C6—H6	120.8	C14—C16—H16A	109.5
C5—C6—H6	120.8	C14—C16—H16B	109.5
C8—C7—C11	109.2 (2)	H16A—C16—H16B	109.5
C8—C7—C4	111.6 (2)	C14—C16—H16C	109.5
C11—C7—C4	111.0 (2)	H16A—C16—H16C	109.5
C8—C7—H7A	108.3	H16B—C16—H16C	109.5
C11—C7—H7A	108.3	C14—C17—H17A	109.5
C4—C7—H7A	108.3	C14—C17—H17B	109.5
N1—C8—C9	120.0 (2)	H17A—C17—H17B	109.5
N1—C8—C7	117.3 (2)	C14—C17—H17C	109.5
C9—C8—C7	122.6 (2)	H17A—C17—H17C	109.5
N2—C9—O2	111.5 (2)	H17B—C17—H17C	109.5
N2—C9—C8	128.2 (2)	N2—C18—H18A	109.5
O2—C9—C8	120.3 (2)	N2—C18—H18B	109.5
C11—C10—O2	122.2 (2)	H18A—C18—H18B	109.5
C11—C10—C15	126.8 (2)	N2—C18—H18C	109.5
O2—C10—C15	110.9 (2)	H18A—C18—H18C	109.5
C10—C11—C12	118.2 (2)	H18B—C18—H18C	109.5
			
C6—C1—C2—C3	1.3 (5)	C7—C8—C9—N2	−169.2 (3)
Br1—C1—C2—C3	−178.6 (2)	N1—C8—C9—O2	−172.9 (2)
C1—C2—C3—C4	−0.1 (5)	C7—C8—C9—O2	11.0 (4)
C2—C3—C4—C5	−1.3 (4)	C9—O2—C10—C11	−4.3 (4)
C2—C3—C4—C7	179.2 (3)	C9—O2—C10—C15	176.5 (2)
C3—C4—C5—C6	1.5 (4)	O2—C10—C11—C12	178.4 (2)
C7—C4—C5—C6	−179.0 (3)	C15—C10—C11—C12	−2.6 (4)
C2—C1—C6—C5	−1.2 (5)	O2—C10—C11—C7	−3.5 (4)
Br1—C1—C6—C5	178.8 (2)	C15—C10—C11—C7	175.5 (2)
C4—C5—C6—C1	−0.3 (5)	C8—C7—C11—C10	12.9 (3)
C5—C4—C7—C8	−69.8 (3)	C4—C7—C11—C10	−110.6 (3)
C3—C4—C7—C8	109.7 (3)	C8—C7—C11—C12	−169.0 (2)
C5—C4—C7—C11	52.3 (3)	C4—C7—C11—C12	67.5 (3)
C3—C4—C7—C11	−128.2 (3)	C10—C11—C12—O4	−177.2 (3)
O1—N1—C8—C9	178.2 (2)	C7—C11—C12—O4	4.6 (4)
O3—N1—C8—C9	−0.4 (4)	C10—C11—C12—C13	1.0 (3)
O1—N1—C8—C7	−5.5 (3)	C7—C11—C12—C13	−177.2 (2)
O3—N1—C8—C7	176.0 (2)	O4—C12—C13—C14	−154.1 (3)
C11—C7—C8—N1	167.3 (2)	C11—C12—C13—C14	27.6 (3)
C4—C7—C8—N1	−69.6 (3)	C12—C13—C14—C17	68.6 (3)
C11—C7—C8—C9	−16.5 (3)	C12—C13—C14—C16	−170.8 (2)
C4—C7—C8—C9	106.6 (3)	C12—C13—C14—C15	−51.9 (3)
C18—N2—C9—O2	2.7 (4)	C11—C10—C15—C14	−23.9 (4)
C18—N2—C9—C8	−177.2 (3)	O2—C10—C15—C14	155.2 (2)
C10—O2—C9—N2	−179.4 (2)	C17—C14—C15—C10	−71.9 (3)
C10—O2—C9—C8	0.5 (3)	C16—C14—C15—C10	167.6 (2)
N1—C8—C9—N2	6.9 (4)	C13—C14—C15—C10	48.5 (3)

### Hydrogen-bond geometry (Å, º)

**Table:**

*D*—H···*A*	*D*—H	H···*A*	*D*···*A*	*D*—H···*A*
N2—H2*N*···O3	0.83 (3)	2.00 (3)	2.618 (3)	130 (3)
N2—H2*N*···O4^i^	0.83 (3)	2.38 (3)	2.969 (3)	129 (3)

Symmetry code: (i) *x*−*y*+1/3, *x*−1/3, −*z*+2/3.
